# Current advances in gene therapy of mitochondrial diseases

**DOI:** 10.1186/s12967-022-03685-0

**Published:** 2022-12-05

**Authors:** Vladislav O. Soldatov, Marina V. Kubekina, Marina Yu. Skorkina, Andrei E. Belykh, Tatiana V. Egorova, Mikhail V. Korokin, Mikhail V. Pokrovskiy, Alexey V. Deykin, Plamena R. Angelova

**Affiliations:** 1 Core Facility Centre, Institute of Gene Biology, Russian Academy of Sciences, Moscow, Russia; 2 Department of Pharmacology and Clinical Pharmacology, Belgorod State National Research University, Belgorod, Russia; 3 Department of Biochemistry, Belgorod State National Research University, Belgorod, Russia; 4 Dioscuri Centre for Metabolic Diseases, Nencki Institute of Experimental Biology, Polish Academy of Sciences, Warsaw, Poland; 5 Laboratory of Modeling and Gene Therapy of Hereditary Diseases, Institute of Gene Biology, Russian Academy of Sciences, Moscow, Russia; 6 Laboratory of Genome Editing for Biomedicine and Animal Health, Belgorod State National Research University, Belgorod, Russia; 7 Department of Clinical and Movement Neurosciences, UCL Queen Square Institute of Neurology, London, UK; 8 Laboratory of Biophysics of Cell Membranes under Critical State, V.A. Negovsky Scientific Research Institute of General Reanimatology, Russian Academy of Sciences, Moscow, Russia

**Keywords:** Mitochondrial diseases, Gene therapy, Energy metabolism, Mitochondrial DNA, Heteroplasmy

## Abstract

Mitochondrial diseases (MD) are a heterogeneous group of multisystem disorders involving metabolic errors. MD are characterized by extremely heterogeneous symptoms, ranging from organ-specific to multisystem dysfunction with different clinical courses. Most primary MD are autosomal recessive but maternal inheritance (from mtDNA), autosomal dominant, and X-linked inheritance is also known. Mitochondria are unique energy-generating cellular organelles designed to survive and contain their own unique genetic coding material, a circular mtDNA fragment of approximately 16,000 base pairs. The mitochondrial genetic system incorporates closely interacting bi-genomic factors encoded by the nuclear and mitochondrial genomes. Understanding the dynamics of mitochondrial genetics supporting mitochondrial biogenesis is especially important for the development of strategies for the treatment of rare and difficult-to-diagnose diseases. Gene therapy is one of the methods for correcting mitochondrial disorders.

**Graphical Abstract:**

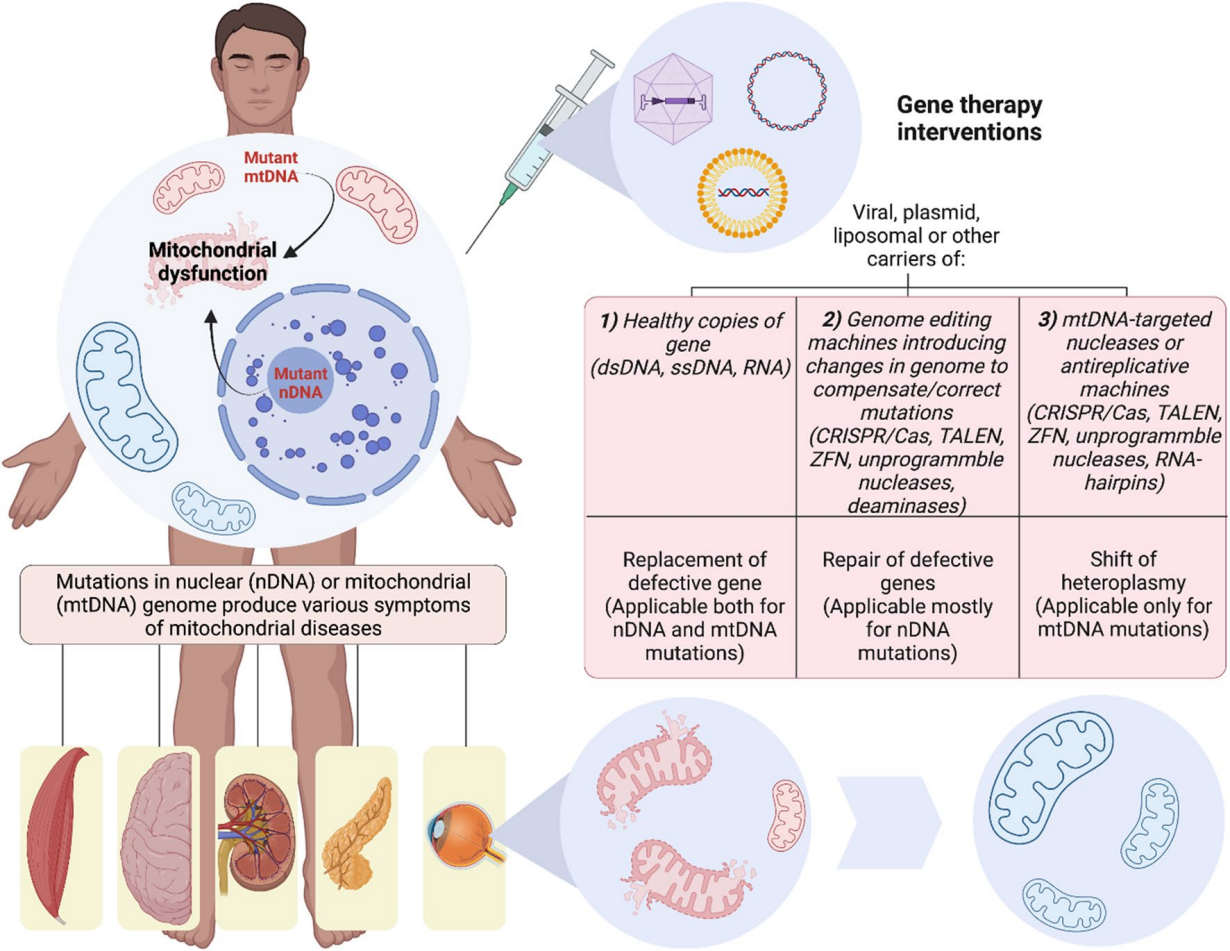

## Introduction

MD—is a heterogeneous group of disorders, caused by mutations in genes, the role of which is crucial for mitochondrial physiology, and for function of the electron transport chain (ETC) and oxidative phosphorylation (OXPHOS), in particular. Causative mutations in MD can occur both in nuclear (n) or mitochondrial (mt) DNA and thus, with exception of rare cases of non-inherited mutations occurring de novo, MD are characterized by monogenic autosomal, X-linked or maternal inheritance [1]. As mitochondria play a key role in metabolism and are involved in a wide spectrum of cellular functions, the mitochondrial dysfunction leads to both tissue-specific and systemic disorders [2]. MD can affect almost all types of tissues, to greater extent the nervous, muscle and retinal [3].

There are still not so many effective ways to enhance mitochondrial function in terms of evidence-based medicine. Currently, drugs targeting MD are primarily antioxidant or metabolic therapy [4], e.g. dichloroacetate (DCA), arginine, coenzyme Q10, idebenone, etc. and their efficiency seems to be very modest [5–7].

Gene therapy (GT) is one of the revolutionary strategies for curing hereditary diseases on which the greatest hopes are so far pinned in this century. The mind-boggling successes shown by the GT drugs for the treatment of spinal muscular atrophy and Leber's optic atrophy are truly impressive and give a reason to hope for further progress. Of special interest is the rapid development of genome-editing systems, particularly *Clustered regularly interspaced short palindromic repeats (CRISPR) / CRISPR-associated protein (Cas)*, which make it possible to precisely repair unwanted mutations [8].

However, because of some aspects of mitochondrial biology, there is a row of stumbling blocks on the way of GT applications in MD. In this review, we focused on the current status including success and difficulties in GT of MD. We critically discuss the possibilities and difficulties of using genome editing systems. Most of all we focused on GT approaches already tested in vivo.

## Basic mitochondrial biology

Mitochondria are organelles responsible for multiple functions in the cells, which consist of two membranes—outer and inner. The inner mitochondrial membrane, containing anchored complexes of the ETC, is the main structure for the mitochondrial respiration and maintenance of the mitochondrial transmembrane potential. Mitochondrial membrane potential is crucial for regulating cellular life and death processes and also serves as a proton motive force in OXPHOS, which generates ATP [9]. Mitochondrial matrix contains cyclic mitochondrial DNA (mtDNA). The transcription and translation of mtDNA differs from the nuclear system and requires more than 100 nuclear-encoded proteins transported into mitochondria by a complex protein transport system [10, 11]. Mitochondria are signaling hubs that integrate the catabolic and anabolic metabolism and regulate cell growth, differentiation, vitality, and death [12]. Mitochondria cannot be synthesised de novo; therefore, they are subjected to constant quality control and regeneration through budding of mitochondrial-derived vesicles and mitophagy [13, 14]. Another important mechanism maintaining stable state of mitochondria is mitochondrial fission and fusion. In brief, mitochondrial fission is essential for growing and dividing cells to populate them with adequate numbers of mitochondria. On the other hand, mitochondrial fusion enables partly impaired mitochondria to physically join together and utilize each other’s protein machinery compensating their own limitations (so called cross-complementation) and maximizing oxidative capacity in response to stress [15].

The mitochondrial genetic system incorporates closely interacting bi-genomic factors encoded by the nuclear and mitochondrial genomes. Genes involved in mitochondrial function are unevenly distributed between the nuclear and mitochondrial genome. Dramatic shift manifests itself in about 1,500 nuclear and just 37 mitochondrial genes. Moreover, circular mtDNA does not contain introns and makes up 1% of the whole cellular DNA. At the same time, mammalian mtDNA is present in thousands of copies per cell and it is characterized by high levels of heterogeneity. It encodes mRNAs for 13 polypeptides of the OXPHOS system, as well as 2 rRNAs and 22 tRNAs for their translation [16]. Four out of five complexes of the ETC and OXPHOS (I, III, IV & V) are composed of subunits, encoded both by the nuclear and mtDNA, while only one (II) is encoded solely by nuclear genome [17].

The mitochondrial genome has a higher mutation rate (about 100–1000 fold), than the nuclear genome [18]. The pathological mutations of mitochondria are survived due to inter-mitochondrial exchange of nutrients and functional complementation taking place through mitochondrial fusion [19, 20]. It leads to heterogeneity of population of mtDNA in single cell or mitochondria and as a result mitochondria become heteroplasmic. There are ~ 1,000 mtDNA molecules in a cell, and in the case of mutations the wild-type mtDNA can compensate for the presence of mutant mtDNA, up to threshold levels, which are usually relatively high, 70–95 [17, 21–23].

## Clinical features of MD

**Fig. 1:**
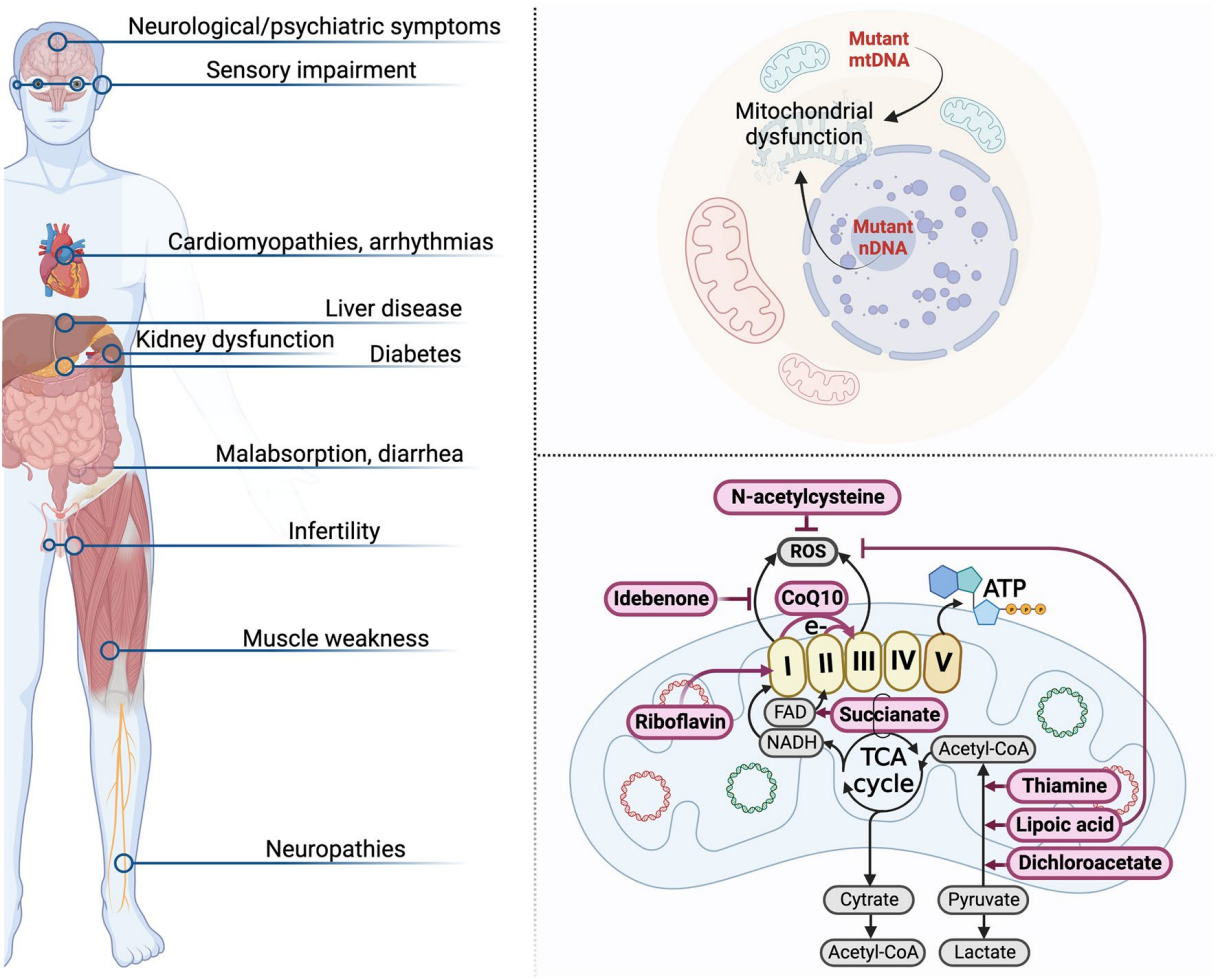
Overview of the MD in the clinical and pharmacological contexts. The patients with MD are characterized by involvement of different organs and tissues which results on various symptoms and their combinations. Of the most typical clinical signs are neurological, sensory and muscular symptoms. All these symptoms are caused by defective work of mitochondrial respiration as a result of mutations in nDNA or mtDNA. Classical pharmacological methods to compensate inadequate functioning of mitochondria rely on reinforcement of mitochondrial metabolic cascades and reduce of toxic agents such as lactate and ROS. For instance, thiamine, lipoic acid and dichloracetate are shown to activate pyruvate dehydrogenase resulting in decrease of lactate accumulation due to turning pyruvate into another metabolite acetyl-CoA. Succinate, riboflavin, and CoQ10 promote ETC donating electrons or restoring the function of complexes I and II. Some compounds such as idebenone, N-acetylcysteine and lipoic acid have the ability to reduce ROS production or inactivate them

Mitochondrial and nuclear mutations lead to ~ 40 clinical forms of mitochondrial diseases (MD) with clear molecular-genetical and biochemical dysfunction of mitochondria. More than 30 of these MD have a mutation in nuclear DNA and more than 10 syndromes and diseases induced by mutations in mtDNA [24]. MD usually affects children, but the age of MD onset, as well as patient’s lifetime, varies a lot. The most common clinical signs of MD include neurological (epilepsy, ataxia, cognitive deficits), sensory (hearing loss, blindness) and muscular (myopathy) symptoms, but the clinical picture can be very different, manifesting in pathology of many other organs (diabetes, liver disease, kidney dysfunction, infertility, arrythmias etc.) (Fig. 1). Some symptoms of MD are prone to manifest together which allows to unite them into syndromes, such as MELAS (Mitochondrial Encephalopathy, Lactic Acidosis, and Stroke-like episodes) or MIDD (maternally inherited diabetes and deafness). Some constellations of symptoms and signs are caused by mutations in different genes and, vice versa, the same mutations can cause different syndromes. Most often, in nearly one-third of the cases, MD are caused by disturbances of the respiratory chain complex I [25–27].

## Standard treatment options for MD

With a limited base of evidence and little data from randomized trials, the treatment of MD remains anecdotal. Moreover, of prescribed treatments the most are considered medical foods [5]. Interventions are mostly focused on vitamin-based and cofactor-based mitochondrial therapies intended to promote critical enzymatic reactions, reduce oxidative stress, and scavenge toxic acyl coenzyme A (acyl CoA) molecules, which accumulate in mitochondrial disease [28]. Using cocktails of vitamins and co-factors is more justified when factors in question are decreased either due to deficiency or defect in their transport [29–31]. Additionally, patients struggling with MD are recommended to diet and lifestyle changes [32, 33].

However, only the small cohorts of patients respond to this treatment since all the methods relying on metabolic therapy are poorly effective because of their non-selectiveness, low mitochondrial hitting and low overlap of their action with disease-causing mechanisms [34, 35]. Standard pharmacological interventions are reviewed in [5, 28, 35]. Some of them are presented in the Fig. 1.

## Methods of gene therapy (GT)

The idea to treat genetic disorders on the genome level as an alternative to the classical pharmacotherapy has been prospering for decades [36]. Actually, it began in the 60s of the previous century, just after several discoveries showed that foreign DNA could be inserted and expressed into mammalian cells [37, 38]. These findings opened up brand-new avenues for medicine, finally resulting in the development of the conception of GT.

GT is “the introduction, removal, or change in the content of a person’s genetic code with the goal of treating or curing a disease” (according to American Society of Gene and Cell Therapy definition) [39]. Classically, GT is based on the delivery of the correct copy of a gene to compensate for the function of the mutated one. It can be either ex vivo treatment of cells (usually for blood cells) collected from patient or in vivo interventions used as systemic or local administration of GT agents.

Physically GT drugs might be viral (adenoviruses, adeno-associated viruses) or non-viral (plasmids, biomaterial particle-based delivery vectors) vectors, containing functional DNA/RNA sequence [40]. Depending on GT strategy these sequences may result in different effects in the cell, to wit: (1) provide the proper version of mutated gene replacing it functionally; (2) modify expression of the mutated gene; (3) change the original sequence of the mutated gene to improve its function.

Replacement therapy is probably most straightforward method of treating monogenic disorders. Once in the cell, the exogenous copy of the correct gene becomes the template for the synthesis of the correct protein, resulting in disease attenuation. Unfortunately, for some monogenic diseases, GT may not be as simple as gene replacement for the disease-causing gene because of dominant disease traits, large gene sizes and immune rejection are a few of the challenges that face gene replacement. In this respect some approaches rely on the use of siRNA (small interfering RNA), shRNA (short hairpin RNA) and miRNA (microRNA) silencing the mutated gene by altering its expression on mRNA level. The last strategy is known as antisense therapy but it is appliable only when causative mutation is displayed as dominant negative or gain-of-function [41].

However, as a matter of fact, the silver bullet for the treatment of monogenic diseases is GT, based on genome editing technologies. It includes approaches based on gene repair involving the correction of an existing mutation to restore the expression of the correct version of the protein [42], Sheila [43]. For precise editing of genome, a few technologies were proposed. Probably the most promising advances in this field are related to CRISPR (Clustered Regularly-Interspaced Short Palindromic Repeats)-Cas9 (CRISPR associated protein 9), the system, which discovery was awarded by 2020 Nobel prize in chemistry. Several CRISPR/Cas9 based in vivo interventions have already reached the first clinical trials [44, 45].

### Zinc fingers nucleases (ZFN) and transcription activator-like effector nucleases (TALEN)

The programmable nucleases ZFN and TALEN contain a variable DNA-binding amino acid sequence and a constant endonuclease domain [46, 47]. Changing the DNA-binding domain by genetic engineering methods, one may target ZFN and TALEN to different regions of the genome, in which nuclease creates double-stranded breaks. To repair these breaks the cell recruits standard DNA repair systems, resulting in homological or non-homological recombination. ZFN and TALEN are recognized as the most efficient genome-editing systems, more precise than CRISPR/Cas9 [48, 49]. However, application of these systems is complicated by difficulties with design of target specific DNA-binding domains: adjacent amino acids influence each other, and the result of this interaction is difficult to predict.

### CRISPR/Cas9

In contrast to ZFN and TALEN, the CRISPR/Cas9 system implements the RNA-mediated recognition of the target DNA. In brief, Cas (Cas9 or others) nucleases are guided by single guide RNA (sgRNA) sequence responsible for specific complementary recognition of target DNA. Endonuclease Cas9 generates a double-stranded break in the DNA molecule, 3–4 nucleotides before motif adjacent to the protospacer (pam-site) [50]. Some modified forms of Cas9 are able to introduce break in only one of the two strands.

Although CRISPR/Cas9 generates more off-target events than TALEN and ZFN, the accuracy of CRISPR/Cas rapidly improves, giving rise to hopes for high clinical efficacy and safety in the future [51].

Detailed description of the ZFN, TALEN and CRISPR/Cas systems can be found in [42, 52].

## GT in the focus of MD

Because of some aspects of mitochondrial biology GT of MD is challenging. First of all, mitochondria are characterized by complicated permeability to all non-mitochondrial proteins, RNA and DNA. Besides that, mitochondrial systems of DNA replication and reparation and even genetic code are different from nuclear ones. Altogether, these hallmarks may not influence the application of GT in MD caused by mutations of nDNA, but trouble treatment of diseases associated with mutations of the mitochondrial genome. Moreover, a cell may contain thousands of mitochondria, each with its own multiple DNA copies and regulatory circuits of gene expression. Finally, for the effective mitochondrial targeting GT-vector must be delivered to all affected tissues. Below we attempt to consider all of these obstacles and current advances on the way of their overcoming.

### Delivery

As mentioned above, the brain, retina and skeletal muscles are the organs most often affected in MD. Multisystem damage can also involve the liver, gastrointestinal tract, pancreas, kidneys, and others. Generation of new GT approach should solve the problems of delivery to these target tissues. Both viral and non-viral delivery systems have been proposed for MD GT. The advantages of non-viral vectors are attracting many researchers to explore the promising delivery system among polymers, lipids, peptides, inorganic materials, and their combinations. The range of application of non-viral vectors is wider due to the possibility of delivering both coding genetic constructs, short, modified oligonucleotides, and large genome editing complexes.

Biodegradable particles demonstrate a perfect safety profile and give the opportunity for repeated delivery. Noncationic liposomes, amphiphile carriers, dequalinium-based liposome-like vesicles (DQAsomes) and others were used for mitochondria targeting in vitro [53, 54]. Current research is focused on increasing transfection efficiency while lowering cytotoxicity [55].

The most popular viral vectors are recombinant adeno-associated viruses (rAAV). They are used in a majority of preclinical and clinical trials for genetic diseases including MD due to their safety profile and widespread biodistribution [56, 57]. Different natural and engineered AAV serotypes effectively deliver DNA constructs into the target tissues and provide long-term expression with a low rate of integration events. Among all known for the time AAV vectors AAV2, AAV5, AAV6, AAV9, AAVrh.10, AAVrh.74 are the most popular serotypes for gene transfer into the brain, eye and skeletal muscles. A detailed review of AAV tropism discovered in NHP studies is presented in Table 1. Natural tropism of viral vectors allows to use of systemic delivery to reach the target site. But other routes of administration that are restricted to the damaged organs become even more popular. Recent research revealed that intracerebrospinal injections of AAV9 or AAVrh10 lead to 10–100 times higher brain expression level than after intravenous injection [58]. Direct gene transfer to the eye during intravitreal (IVT), suprachoroidal (SC), or subretinal (SR) injections demonstrate robust transgene expression in target cells. SR administration was most efficient in targeting the retina and photoreceptors [59]. Percutaneous transendocardial delivery helps to transduce heart tissue by various AAV serotypes [60, 61]. Additional technologies can promote transduction. For example, MRI-guided focused ultrasound (MRIgFUS) helps AAV6 to pass BBB and transduce neuronal cells without accumulation in the liver [62]. New serotypes of AAV are intensively tested in preclinical trials. They are designed to increase the specificity and effectiveness of delivery. The rapid evolution of AAV9 resulted in new variants AAV-PHP.eB and 9P03-33 with enhanced tropism to the central nervous system and lowered liver transduction [63, 64].

**Table 1:** AAV serotypes biodistribution demonstrated in non-human primates’ studies

Organ	Serotype	References
Brain	AAV2	[66], [67], [68]
	AAV5	[69]
	AAV8	[70]
	AAV9	[58], [71], [72]
	AAVrh.10	[58], [73], [74], [75], [76]
Retina	AAV5, AAV2-HBKO	[77]
	AAV2.NN, AAV2.GL	[78]
	AAV9	[79]
Skeletal muscles	AAV1	[80]
	AAV8	[70], [80]
	AAV9	[80]
	AAVrh.74	[81]
Heart	AAV1	[80]
	AAV6	[61]
	AAV8	[61]
	AAV9	[61, 80]
	AAVrh.10	[58]
	AAVrh.74	[81]
Liver	AAV2, AAV5, AAV7, AAV8	[82]
	AAV3B	[83]
	AAV9	[58]
	AAVrh.10	[58]
Pancreas	AAV2	[66]
	AAV7	[84]
Kidney	AAV3B	[83]
	AAV2, AAV5, AAV7, AAV8	[82]
	AAVrh74	[81]
Gastro-intestinal tract	AAV6, AAV8, AAV9	[61]
	AAV2	[66]
	AAV5	[82]

Advances in AAV-vector based therapy of MD are summarized in the recent review [65].

### Mitochondrial transfer of GT components

Deficiency in mitochondrial proteins or RNA could be compensated only by direct mitochondrial targeting of gene therapeutic agents. Considering that, inner membrane of mitochondria is mainly impermeable and amino acids or nucleotide resides should be delivered to the matrix of mitochondria using special natural or artificial transporters.

#### Proteins

Mitochondrial proteins, encoded in the nuclear genome, could be transferred to the mitochondria by N-terminal presequences that serve as mitochondrial targeting sequences (MTSs) and are cleaved upon import [85, 86]*.* These sequences form amphipathic helices that vary largely in primary sequence but are characterized by a length of about 15–60 residues, a net charge of + 3 to + 6, the absence of negatively charged residues and a high content of hydroxylated amino acids [86, 87]. Rational design methods have also been utilized for the creation of new viral capsids with penetrating peptides or mitochondria localization signals [88, 89].

#### RNA

Mitochondrial import of RNA is a sophisticated and not entirely clear process. Mammals harbor 3 specific types of non-coding RNA transferring into mitochondria after synthesis in the nucleus [90]. In addition, some authors [91, 92] reproduced the mechanism of mitochondrial transfer of tRNA in mammals which was previously identified only for yeasts [93]. This yeast-specific translocation of tRNA is mediated by a protein-import-like mechanism and its crucial components are F- and D-hairpins in RNA structure [94].

The ability for transfer other types of RNA into mitochondria is still under question. mRNA can only be localized on the outer layer [95, 96], but does not pass through the mitochondrial membrane. This surface mitochondrial localization is guided by specific mitochondrial targeting sequences in 5ʹ prime and 3’UTR. Being translated from mitochondrial surface-anchored mRNA, mitochondrial proteins are immediately transported into mitochondria. This was shown, in particular, for the expressed in the nucleus mitochondrial *ATP6* gene (provided with MTS) and the 3′UTR of the nuclear *SOD2* (superoxide dismutase 2) gene 3′UTR of [97].

Most studies of mitochondrial RNA transfer conclude that RNA can be translocated only by engaging protein import mechanisms [98]. In this way, after extramitochondrial assembly the ribonucleoprotein complex can be translocated into mitochondria [99]. In terms of GT of MD, this opens avenues for mitochondrial delivery of RNA-based therapeutic agents such as CRISPR/Cas or antireplicative machines such as FD-RNA (see below).

#### DNA

Several strategies of direct mitochondrial gene delivery were proposed. This might be DNA delivery by MTS-harbouring vectors, modified delivery conditions, reversed charge, etc. [100]. For instance, the use of mitochondrially-targeted AAV was shown to be effective in the mitochondrial transfer of mutant human *ND6* gene in murine mitochondria [101].

Another interesting approach for targeting mitochondria was proposed by Yasuzaki et al. In the procedure, called “hydrodynamic injection”, a large volume of naked plasmid DNA is rapidly injected, resulting in plasmids-to-mitochondrial transfer [102].

Yamada et al. described “MITO-Porter”, a liposome-based carrier that introduces cargos into mitochondria via a membrane fusion mechanism. The system consists of high-density octaarginine-modified liposomes which can escape from macropinosomes efficiently to the cytosol keeping the encapsulated compounds intact. Upon release from the macropinosomes, MITO-Porter then binds to the mitochondrial membrane via electrostatic interactions, which induce fusion between the MITO-Porter and the mitochondrion [103]. MITO-Porter-based systems were shown to be effective in the mitochondrial transfer of DNA as well as for proteins and RNA[104–107].

Detailed description of the advances in mitochondria-targeted therapeutics delivery is presented in [53, 54, 108].

## GT of diseases caused by nDNA mutations

In general, GT of MD caused by nDNA mutations completely corresponds GT strategies used in the treatment of not-mitochondrial monogenic diseases. In the case of these disorders there is no need to overcome difficulties determined by peculiarities of mitochondrial biology such as low mitochondrial transfer of GT-components, mismatch of genetic code between mitochondrion and nucleus and others (the problems with mtDNA are discussed in the "GT of diseases caused by mtDNA mutations" section in details).

The most prevalent strategy for the treatment of MD, associated with altered nucleus-encoded genes is the simple transport of copies of proper DNA into the affected cells. Fortunately, there are various animal models of MD [109] in which such an approach was tested (summarized in Table 1).

### TYMP

Mitochondrial neurogastrointestinal encephalopathy (MNGIE) syndrome is a rare autosomal recessive MD, caused by mutation in *TYMP*, the nuclear gene encoding the enzyme thymidine phosphorylase (TP) [110]. In patients, TP dysfunction causes accumulation of the nucleosides—thymidine and deoxyuridine, which interferes with mtDNA replication [111, 112].

In MNGIE murine model with double *Tymp/Upp1* knockout, Torres-Torronteras et al. demonstrated, that administration of haematopoietic donor cells with *TYMP* coding sequence transduced by lentiviral vector, allows to restore TP activity and to decrease metabolic abnormalities [113]. Later, the same group evaluated in vivo approaches of *TYMP* replacement therapy via systemic administration of AAV containing the *TYMP* coding sequence transcriptionally targeted to the liver [114, 115]. In both studies, AAV-mediated GT appeared to be effective in the correction of biochemical abnormalities.

Ferran Vila-Julià et al. enhanced clinical phenotype in the MNGIE mice by administration of thymidine and deoxyuridine and then tested several doses of AAV8 vector carrying the human *TYMP* coding sequence under the control of different liver-specific promoters (TBG, AAT, or HLP). In this study different gene therapeutic agents ameliorated both biochemical and clinical phenotype in a dose-dependent manner [116].

### Slc25a46

In another study Li Yang et al. tested GT in mice carrying a knockout of the mitochondrial fusion-fission-related gene solute carrier family 25 member 46 (Slc25a46). They showed AAV-Slc25a46 treatment was able to rescue the premature death in the Slc25a46-/- mice, restoring mitochondrial complex activities, normalizing mitochondrial morphology, and improving electrical conductivity of the nervous system, as well as attenuating neurodegeneration and neuroinflammation [117].

### OPA1

Several successful gene therapeutic approaches based on AAV-mediated delivery of *OPA1* gene were proposed. *OPA1* encodes a mitochondrial enzyme called dynamin-like 120 kDa protein. This is a large mitochondrial GTPase with crucial roles in membrane dynamics and cell survival [118]. *OPA1* mutations affect mitochondrial fusion, energy metabolism, control of apoptosis, calcium clearance and maintenance of mitochondrial genome integrity. *OPA1* mutations can cause dominant Optic Atrophy (DOA), a neuro-ophthalmic condition characterized by bilateral degeneration of the optic nerves, causing insidious visual loss, typically starting during the first decade of life [119, 120].

In a murine model of DOA, caused by mutation c.2708_2711delTTAG in *Opa1* gene, IVT injection of AAV2 carrying the human *OPA1* cDNA under the control of the cytomegalovirus promoter resulted in the prevention of retinal degeneration [121].

*OPA1* GT was also assessed in non-genetic models. Rotenone is a lipophilic, naturally occurring compound acting as a strong inhibitor of complex I of the mitochondrial respiratory chain [122]. Using the murine model of rotenone-induced optic neuropathy, Maloney et al. the reported therapeutic benefit of OPA1 isoform 1 and 7 delivered by AAV-2 via IVT route [123].

In another study AAV-mediated OPA1 delivery was used to ameliorate retinal damage caused by ischemia–reperfusion (I/R). AAV–Opa1-ΔS1, IVT injected 25 days before retinal I/R injury, significantly prevented I/R-induced retinal thinning and the cell loss in the ganglion cells layer, reducing levels of markers of apoptosis and necrosis [124].

Finally, AAV-mediated delivery of OPA1 was tested to attenuate neurological condition in a rat model of cerebral focal I/R. It was shown that intracranial injection of AAV-OPA1-v1ΔS1 markedly improved cerebral I/R-induced motor function damage, attenuated brain infarct volume, neuronal apoptosis, mitochondrial bioenergetics deficits, oxidative stress, and restored the morphology of mitochondrial cristae and mitochondrial length. It also preserved the mitochondrial integrity and reinforced the mtDNA content and expression of mitochondrial biogenesis factors in ischemic rats [125].

### NDUFS4

Mutations in *NDUFS4* gene, encoding NADH dehydrogenase (ubiquinone) iron-sulfur protein 4, result in compromised activity of mitochondrial complex I, causing Leigh syndrome- the most common infantile mitochondrial encephalopathy [126]. Several studies have shown that administration of human *NDUFS4* coding sequence by AAV2/9 and/or AAV-PHP.B vectors improved clinical phenotype and prolonged the lifespan in *Ndufs4* − / − mice representing a model of Leigh syndrome [127–129].

### Fdxr

Li Yang et al. have utilized a mouse model carrying a p.Arg389Gln mutation of the mitochondrial Ferredoxin Reductase gene (*Fdxr*) and used it to test neurotropic AAV-PHP.B vector loaded with the mouse *Fdxr* cDNA sequence. They observed that the AAV vector was effectively transduced in the central nervous system and all peripheral organs, and AAV-Fdxr treatment reversed almost all the symptoms of the mutants. This therapy improved the electrical conductivity of the sciatic nerves, prevented optic nerve atrophy, improved mobility, and restored mitochondrial complex function [130].

### ETHE1

Ethylmalonic encephalopathy (EE) is a severe monogenic disorder caused by mutations of the nuclear gene *ETHE1*, encoding a ubiquitous mitochondrial protein sulfur dioxygenase (SDO) (note: EE is not classified as MD) [131], involved in the detoxification of H_2_S [132, 133] showed that AAV2/8-mediated, *ETHE1*-gene transfer to the liver of a genetically, metabolically, and clinically faithful EE mouse model resulted in the full restoration of SDO activity, correction of plasma thiosulfate, a biomarker reflecting the accumulation of H_2_S, and spectacular clinical improvement [133].

Descriptions of GT approaches based on the replacement of mutated mitochondrial proteins encoded in the nucleus are summarized in Table 2.

**Table 2:** Tested approaches for delivery of nuclear genes associated with MD

Delivered gene	Description of model	Vector and route of administration	Effect	Reference
Full cDNA of human wild-type *TYMP*	Mitochondrial neurogastrointestinal encephalopathy syndrome (MNGIE) murine model (Double Tymp/Upp1 knockout) Represents nDNA mutation	Single intravenous (IV) administration of haematopoietic donor-derived cells with *TYMP* coding sequence transduced by lentiviral vector	Restoration of thymidine phosphorylase (TP) activities in peripheral blood cells of treated mice Decrease of plasma thymidine and deoxyuridine concentrations to levels in the range of wild-type mice	[113]
Full cDNA of human wild-type *TYMP*	Mitochondrial neurogastrointestinal encephalopathy syndrome (MNGIE) murine model (Double Tymp/Upp1 knockout) Represents nDNA mutation	Single IV injection (tail vein) of AAV2/8-TBG-hcTYMP	Dose-dependent restoration of TP activity in the liver and improvement of biochemical abnormalities in the liver and blood (34 weeks after treatment)	[114],
Full cDNA of human wild-type *TYMP*	Mitochondrial neurogastrointestinal encephalopathy syndrome (MNGIE) murine model (Double Tymp/Upp1 knockout) Represents nDNA mutation	Single IV injection (tail vein) of AAV2/8-TBG-hcTYMP	Dose-dependent restoration of TP activity in the liver and improvement of biochemical abnormalities in the liver and blood (21 months after treatment)	[115]
Full cDNA of human wild-type *TYMP*	MNGIE murine model (Double Tymp/Upp1 knockout + chronic oral administration of thymidine and deoxyuridine)	Single IV injection (tail vein) of liver-targeted AAV vectors (AAV-TBG, AAV-AAT, or AAV-HLP)	Restoration of TP activity in liver Amelioration of biochemical abnormalities Improvement of motor functions AAV-AAT and AAV-HLP treatment prevented ventricular enlargement Normalization of the mitochondrial dNTP balance	[116]
cDNA of mouse wild-type *Slc25a46*	*Slc25a46* knockout mice	Single IV injection (facial vein of pups) of neurotrophic AAV–PHP.B vector	Prolongation of lifespan Increase of bodyweight Attenuation of central nervous system defects and ataxia Attenuation of optic atrophy Restoration of mitochondrial morphology and activity in various tissues	[117, 130]
cDNA of human wild type *OPA1*	Hemizygous OPA1 ± mice carrying human OPA1 transgene with c.2708_2711delTTAG mutation	Single IVT injection of AAV2 serotype 2 vector	Modest restoration of visual acuity Prevention of loss of retinal ganglion cells number	[121]
cDNA of codon-optimized versions of human *OPA1* isoform 1 and 7	Wild type mice with rotenone-induced retinal degeneration	Single IVT injection of AAV2 serotype 2 vector	Improvement of spatial visual function	[123]
cDNA of Opa1 long isoform with 11 residues (190–200) deleted in the S1 cleavage site (Opa1-ΔS1)	Rats with ischemia–reperfusion retinal injury	Single IVT injection of AAV	Normalization of the ischemia–reperfusion-induced downregulation of β-tubulin ΙΙΙ and Brn3a Inhibition of the retinal thickness and the cell loss in the ganglion cells layer Attenuation of elevation of receptor-interacting protein 3 and cleavage of caspase 3	[124]
cDNA of human *OPA1* long isoform with deletion of the S1 cleavage site (OPA1-v1ΔS1)	Rats with focal cerebral ischemia–reperfusion injury	Single IVT injection of AAV	Decrease of neurological deficit and attenuation of infarct volume Restoration of mitochondrial cristae morphology and mitochondrial length Preservation of mitochondrial integrity	[125]
Human cDNA of wild-type *NDUFS4*	Constitutive Ndufs4 − / − mouse model developing a rapidly progressive encephalopathy, starting ~ 40 days after birth	IV or intracerebroventricular (ICV) or IV + ICV injections of AAV2/9	Systemic AAV2/9-hNDUFS4 restores complex I assembly and activity in peripheral tissues but does not ameliorate the clinical phenotype ICV injections of AAV2/9-hNDUFS4 slightly ameliorate the clinical phenotype in newborn Ndufs4 − / − mice Double IV + ICV injections in newborns of AAV2/9-hNDUFS4 ameliorate the clinical phenotype and increases lifespan of Ndufs4 − / − mice	[127]
Human cDNA of wild-type *NDUFS4*	Constitutive Ndufs4 − / − mouse model developing a rapidly progressive encephalopathy, starting ~ 40 days after birth	Single IV (tail vein in adult mice, temporal vein in newborns) of AAV-PHP.B	Prolongation of the lifespan, improvement of motor functions and complex I assembly in adult mice Absence of effect and typical disease progression in newborns	[128]
Human cDNA of wild-type *NDUFS4*	Constitutive Ndufs4 − / − mouse model developing a rapidly progressive encephalopathy, starting ~ 40 days after birth	Single IV injection (retroorbital sinus)	Increase of survival rate and body weight Improvement of motor functions Prevention of neuronal and glial pathology Improvement of retinal function	[129]
cDNA of murine wild-type *Fdxr*	Fdxr R389Q/R389Q mice	IV injections of AAV-PHP.B vector (temporal facial vein of neonatal mice)	Alleviation of neuronal gliosis and neurodegeneration in the CNS Mitigation of the optic atrophy, reduction of the movement disorders and sensory neuropathy Improvement of mitochondrial function, decrease of iron overload	[117, 130]
cDNA of human wild-type *ETHE1*	*Ethe1 -/-* mice	Intra-cardiac injections of AAV2/8	Increase of survival rates and body weight Attenuation of biochemical abnormalities	[133]
cDNA of human *TK2*	*Tk2*^*KI*^ mice	Single IV injection of AAV9 or sequential IV injection of AAV9 and AAV2	enhanced replacement therapy with pyrimidine deoxynucleosides delaying disease onset and extending lifespan in mice	[136]

### TK2

Autosomal recessive human thymidine kinase 2 (TK2) mutations cause TK2 deficiency, which typically manifests as a progressive and fatal mitochondrial myopathy in infants and children (note: not classified as MD). Normally, TK2 localizes to the mitochondria and specifically phosphorylates thymidine, deoxycytidine, and deoxyuridine, required for mtDNA synthesis. Decrease in TK2 function leads to mtDNA depletion resulting in mitochondrial dysfunction [134].

Krishnan S. et al. showed attenuation of mtDNA depletion in TK2-deficient mice transgenic with nucleoside kinase from *Drosophila melanogaster* (*Dm-dNK*). In these *Dm-dNK*^±^*Tk2*^*−/−*^ the lifespan was expanded for 3 weeks to at least 20 months and the reduction of subcutaneous and visceral fat was the only visible difference compared with wild type mice [135].

TK2-caused mitochondrial defects in murine model was also compensated via gene therapeutic approach based on AAV9 delivery of human *TK2* cDNA. This intervention enhanced replacement therapy with pyrimidine deoxynucleosides delaying disease onset and extending lifespan in mice expressing mutant TK2 (*Tk2*^*KI*^). Furthermore, sequential treatment of *Tk2*^*KI*^ mice with AAV9 first followed by AAV2 at different ages enabled to reduce the viral dose while further prolonging the lifespan [136].

## GT of diseases caused by mtDNA mutations

**Fig. 2:**
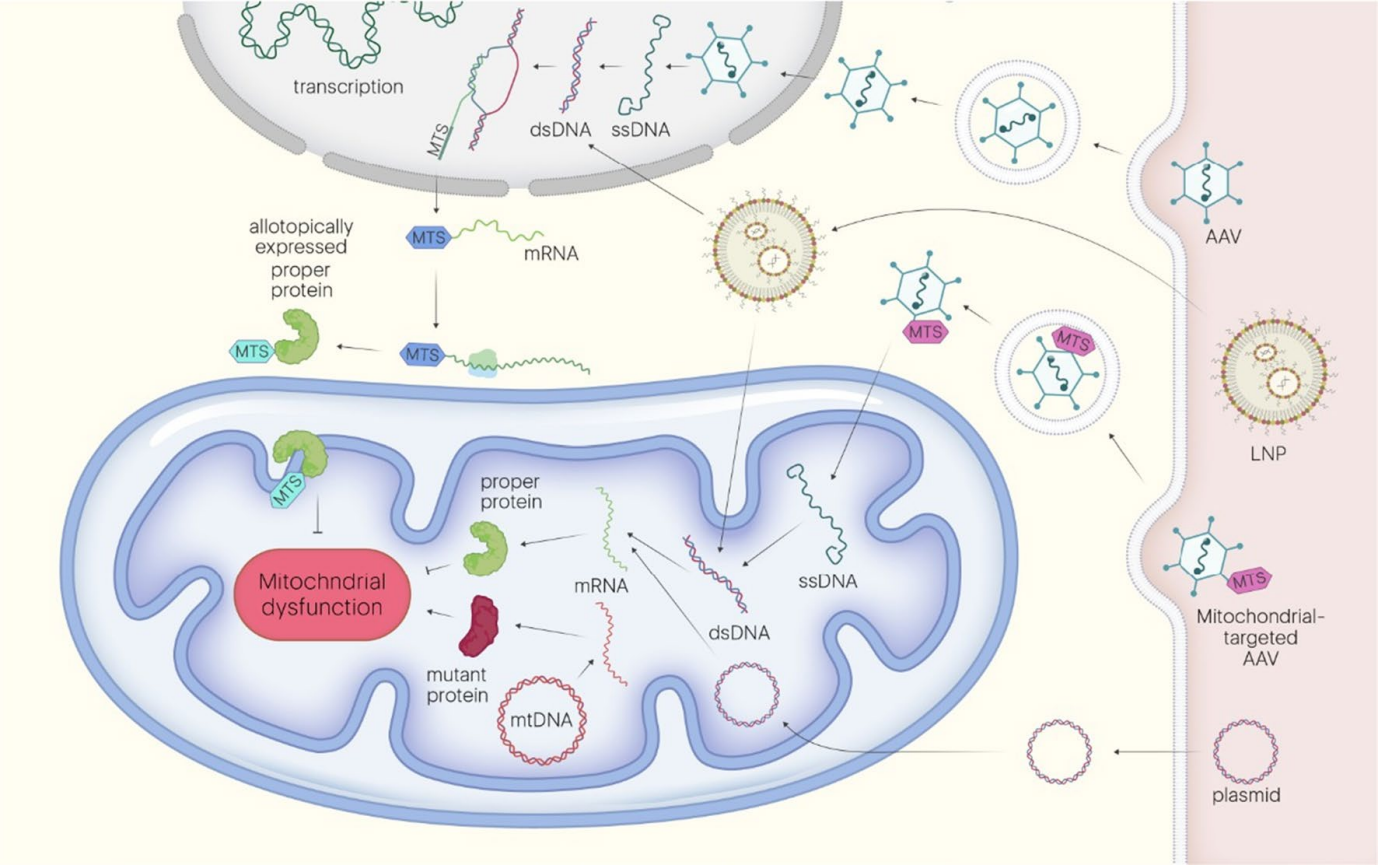
Basic approaches for replacement of causative for MD genes with the use of allotopic expression or direct mitochondrial transfer. In order to compensate the function of altered mitochondrial gene, the delivery of correct forms of DNA or mRNA is used. The sequence is transferred in the form of plasmid or delivered by AAV, LNP or in. **1.** AAV vectors are able to deliver single-stranded (ss)DNA in nucleus where it further replicates to form double-stranded (ds)DNA, template for synthesis of the proper version of mitochondrial protein. To guide this protein into mitochondria coding ssDNA must contain mitochondrial targeting sequence (MTS). **2.** AAV-MTS vectors carrying MTS on its surface are able to directly target mitochondria to provide mitochondrial transfer of ssDNA and subsequent synthesis of proper protein with the use of mitochondrial apparatus. **3.** dsDNA can be delivered by being packed in lipid nanoparticles (LNP). As the part of LNP dsDNA apparently can be targeted directly into mitochondria or allotopically expressed in the nucleus, **4.** The protein’s coding sequence is delivered into the mitochondria in the form of plasmid by hydrodynamic injection

**Fig. 3:**
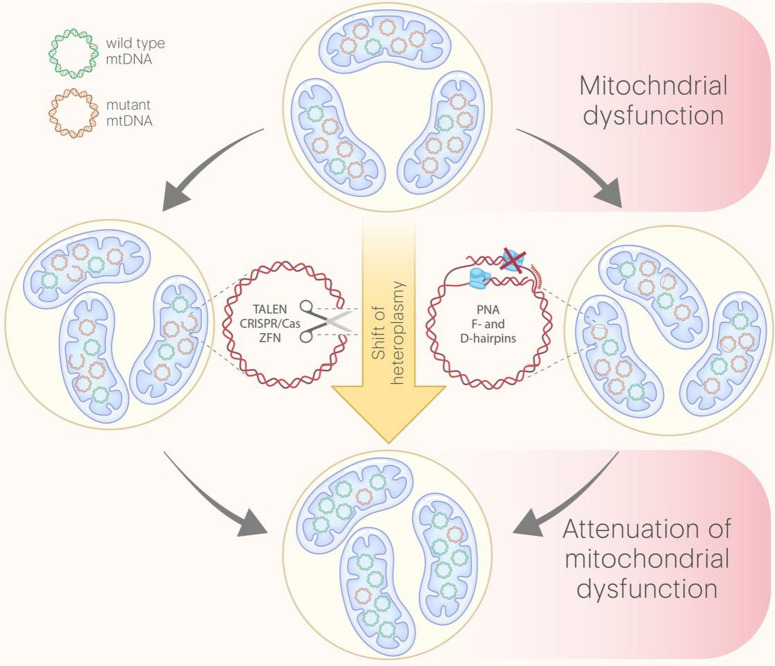
Representation of the basic strategies allowing for decrease of the mutant mtDNA content. The ratio between mutant and wildtype mtDNA (heteroplasmy) determines the severity of mitochondrial dysfunction. One way (left) for shifting heteroplasmy is based on the introduction of double strand breaks (DSBs). To introduce DSBs the nuclease machineries (TALEN, ZFN or CRISPR/Cas) targeted on mutant loci are used. Since all the linear dsDNA molecules are normally being eliminated, the levels of mutant mtDNA decrease. Another way (right) to shift heteroplasmy is to complicate mtDNA replication and hence decrease its content against wild type mtDNA copies. Peptide nucleic acid oligomers (PNC) and F-/D-hairpins containing RNA (FD-RNA) can directly pair with high affinity to the sequence of interest and interrupt further replication of this region. Both ways lead to shift of heteroplasmy resulting in attenuation of mitochondrial dysfunction

In contrast to nDNA, targeting mtDNA mutations determines the necessity to search for original strategies taking into account unique traits of mitochondrial genome organization. If one is aimed to replace mutated gene encoded by mtDNA, such factors as difficulties with mitochondrial transfer of GT components and mitochondrial genetic code must be considered. In the case of gene editing strategies, circularity of mtDNA, differences in nDNA and mtDNA reparation mechanisms (DSBs in mtDNA cannot be repaired) and once again selectiveness of mitochondrial permeability may trouble applicability of standard methods. However, for diseases caused by mutations in mtDNA, several methods, including allotopic expression, direct mitochondrial transfer, replacement of mitochondrial proteins by orthologs, base edition were proposed and successfully tested in vivo ("Allotopic expression of mitochondrial genes", "Replacement of mitochondrial proteins by orthologs", "Direct mitochondrial transfer of mitochondrial genes" sections and Fig. 2). Interestingly, unique mechanisms of mtDNA reparation enable to use nuleases to selectively remove mutant mtDNA ("Shift of heteroplasmy" section and Fig. 3).

### Allotopic expression of mitochondrial genes

Allotopic expression stands for “mitochondrial gene transfer to the nucleus”. Before the transfer, mitochondrial genes should be re-coded into nuclear code and re-targeted to mitochondria by addition of MTS coding sequence. Interestingly, MTS can direct both translated protein and mRNA to mitochondria. In the second case translation occurs on the outer layer of mitochondria but with the use of classic cytoplasmic mRNA (Detailed description in review [137]). Notably, polypeptides encoded by mtDNA are highly hydrophobic, this may cause problems such as aggregation of the expressed polypeptides in cytoplasm and triggering immune response by over-expressed hydrophobic subunits in the cytoplasm [138]. For instance, mitochondrial proteins encoded in nDNA require chaperones to keep them soluble in the cytosol [139].

The strategy of allotopic expression as a roundabout route for GT of MD was proposed in 2002 by G. Manfredi et al. [140] to treat 8993 T– > G mutation of mtDNA MTATP6 gene causing impaired mitochondrial ATP synthesis in two related MD: neuropathy, ataxia, and retinitis pigmentosa and maternally inherited Leigh syndrome. The authors transfected wild-type mitochondrial MTATP6 gene (recoded to be compatible with the universal genetic code and appended to MTS and carboxy-terminal FLAG epitope tag) in the nucleus of wild-type and mutant human cells. After transfection of wild-type human cells, the precursor polypeptide was expressed, imported into, and processed within mitochondria, and incorporated into complex V. Allotopic expression of stably transfected constructs in cytoplasmic hybrids (cybrids) homoplasmic with respect to the 8993 T– > G mutation showed a significantly improved recovery after growth in the selective medium as well as a significant increase in ATP synthesis [141].

In vivo delivery of allotopically expressed genes was successfully tested in several in vivo studies in rodents. Qi et al. have developed an approach for modeling of LHON in mice by translocation of a nuclear version of the human ND4 subunit of complex I (naturally encoded in mitochondrial genome) carrying human mutation Arg340His into the mitochondria of rodent RGCs due to AAV-2 vector. This amino acid substitution caused by AG-to-A transition at nucleotide 11778 in mtDNA is responsible for half of the cases of Leber hereditary optic neuropathy (LHON), a disease that causes blindness in young adults [142, 143]. Allotopic expression of mutant human ND4 in the mouse visual system disrupted mitochondrial cytoarchitecture, elevated reactive oxygen species, induced swelling of the optic nerve head, and induced apoptosis, with a progressive demise of ganglion cells in the retina and their axons comprising the optic nerve [144].

S. Ellouze et al. created a similar model of LHON representing Arg340His mutation of ND4 in rats [145]. The authors expressed mutant human mitochondrial gene ND4 in the retinal cells of rats via electroporation and showed development of retinal abnormalities similar to the classic course of LHON [145]. The treatment induced visual abnormalities and degeneration of retinal ganglion cells, which were 40% less abundant in treated eyes than in control eyes. A subsequent electroporation with wild-type ND4 prevented both retinal ganglion cells loss and the impairment of visual function [145].

J.Guy et al. allotopically expressed wild-type human ND4 complex I subunit in mice, showing its effective mitochondrial delivery and safety for the mouse visual system [146]. Human ND4 was properly processed and imported into the mitochondria of RGCs and axons of mouse optic nerve after IVT injection. The expression of normal human ND4 in murine mitochondria did not induce the loss of RGCs, ATP synthesis, or pattern electroretinography amplitude, suggesting that allotopic ND4 may be safe for the treatment of patients with LHON.

The only clinically tested approach of treatment of MD was based on allotopical expression—nuclear transfer of ND4 coding sequence by IVT application of AAV2. Re-coded in the “universal” genetic code ND4 with appended targeting sequence derived from the P1 isoform of subunit c of the ATP synthase (ATPc) for import into the mitochondrion (rAAV2-ND4), it was shown to be effective in LHON patients [147–149]. IVT injections of AAV2/2-ND4 led to progressively improved visual acuity in Phase 3 clinical trials [150].

Moreover, there is evidence that mitochondrial tRNAs can also be expressed allotopically. Using F- и D-hairpins-dependent mechanism of transport of tRNA, MERRF syndrome [91] as well as MELAS syndrome (A3243G mutation of mitochondrial tRNA) [92] were corrected in human cell models.

### Replacement of mitochondrial proteins by orthologs

Another elegant GT strategy is based on the use of small orthologs of human ETC components, much more compact and encoded by single non-mammalian genes. For instance, transkingdom GT for complex I diseases was proposed for NDI1, yeast ortholog of human NADH dehydrogenase. In the S. cerevisiae mitochondria, the NDI1 enzyme catalyses electron transfer from NADH to Q in the matrix compartment and is the main entry point into the respiratory chain, just as in complex I. The yeast NDI1 gene is composed of 1539 bp which is predicted to encode a precursor polypeptide of 513 amino acid residues. The first 26 amino acid residues of the N-terminal serve as signal sequence for import into mitochondria [151]. In a few studies NDI1 gene of *Saccharomyces cerevisiae* was successfully introduced into mammalian cell lines. The expressed protein was correctly targeted to the matrix side of the inner mitochondrial membranes and was fully functional and restored the NADH oxidase activity to the complex I-deficient cells. The transduced cells were more resistant to complex I inhibitors and diminished production of reactive oxygen species induced by rotenone [151–155].

Chadderton et al. treated mice with rotenone-induced mitochondrial dysfunction by intraocular injection of a nuclear NDI1. In this study, recombinant AAV2 expressing NDI1 (AAV-NDI1) was shown to protect RGCs in a rotenone-induced murine model of LHON, significantly reducing RGC death by 1.5-fold and optic nerve atrophy by 1.4-fold [156]. Authors suggest a clear advantage of using the NDI1 gene over allotopic expression of mammalian mitochondrial complex I subunit genes as a therapy for LHON and that a single gene may provide benefit to all LHON patients with a complex I deficiency irrespective of which complex I subunit gene is causative of this debilitating retinopathy.

### Direct mitochondrial transfer of mitochondrial genes

Hong Yu et al. recapitulated LHON-associated phenotype by mitochondrial transfer of the mutant human NADH ubiquinone oxidoreductase subunit VI gene with T14484C mutation (hND6T14484C) in retinal ganglion cells of adult mice. In brief, via IVT injection of mitochondrial-targeted AAV, *hND6T14484C* was directly delivered into mitochondria causing retinal thinning, apoptosis in RGCs and visual loss. Immunostaining of the retinal slices showed sufficient expression of GT-components colocalizing with a mitochondrion-specific dye MitoTracker Deep Red [101]. This work brightly demonstrates that in vivo methods of direct delivery of gene therapeutic agents into mitochondria work.

Yasuzaki et al. demonstrated effective transfer of exogenous DNA to mitochondria in skeletal muscle of rats following hydrodynamic limb vein injection. In brief, in the hydrodynamic limb vein injection procedure a tourniquet is used to limit the delivery area to one limb per injection and naked pDNA is rapidly injected into the vein in the anterograde direction [157]. It was shown that after hydrodynamic injection of pcDNA3.1 ( +)-luc plasmid, plasmid DNA had been localized in isolated mitochondria of the limbs [102].

### Shift of heteroplasmy

Heteroplasmy, the amazing feature of mitochondria, is another key to the treatment of MD. Heteroplasmy is a dynamically changing result of co-existence of diverse types of mtDNA in one organism. One way to shift heteroplasmy from mutant to wild type of mitochondria is to eliminate mutated mtDNA without any precise editing. This approach involves targeting mutation resulting in non-repairable disruption or inhibition of replication in mutant mtDNA. As a result, wild type mtDNA copies begin to prevail which leads to attenuation of mitochondrial dysfunction. Notably, each mitochondrion most often contains both mutant and wild type mtDNA [158] so elimination of mutant mtDNA could scarcely cause mitochondrial death [159].

#### Antireplicative machines

The first approach to shift heteroplasmy was the application of peptide nucleic acid (PNA) oligomers interrupting replication of mutant mtDNA [160]. These small molecules, where the individual nucleobases are linked to an achiral peptide backbone [161], have greater affinity than equivalent oligodeoxynucleotide when pairing with single stranded complementary DNA. Moreover, PNA is not charged and have has increased cellular and, apparently mitochondrial permeability, especially after small modifications [162]. The basic principle of PNA-dependent heteroplasmy shift is in inhibition of mtDNA replication after strong binding with mutant-specific mtDNA sequence (Fig. 3). This mechanism is feasible because mtDNA is single-stranded during much of its replication [163].

Similarly, there were attempts to design specific antireplicative RNA molecules, complementary to mutant mtDNA [164]. To guide these RNAs into mitochondria they were added by F- and D-hairpins, the structures providing efficient mitochondrial transfer (see "Mitochondrial transfer of GT components" section). The resulting FD-RNAs were successfully tested in Kearns–Sayre syndrome, caused by 4.9 kb-lenght deletion in the mitochondrial genome [165] and in cybrid cells carrying pathogenic A13514G point mutation in the mtDNA ND5 gene [166].

#### Nucleases

Apparently, in contrast to mitochondria of plants [167], and probably fishes [168], mammalian mitochondria not only cannot repair double strand breaks (DSBs), but also have systems of rapid degradation of double stranded linear DNA molecules [169–172]. Since double-stranded breaks cause degradation of mtDNA, the nucleases can be used to eliminate mtDNA molecules (Fig. 3). For the first time, the approach based on the use of high-specific nucleases to shift heteroplasmy was proposed in 2001. Srivastava and colleagues showed that bacterial enzyme PstI endonuclease which has two restriction sites in the human genome can decrease the levels of mutant mtDNA being expressed in mammalian cells [173]. One of these restriction sites is specific for patients with neuropathy, ataxia and retinitis pigmentosa (NARP), as the T8399G mutation creates a unique restriction site that is not present in wild-type human mtDNA. However, not surprisingly, the only application of PstI is limited by patients carrying this particular mutation. The same limitations are specific to all non-programmable nucleases.

##### TALEN and ZFN

Minczuk et al. developed an approach for routing engineered ZFNs to mitochondria, constructing chimeric enzymes targeted to specific mtDNA sequences. A basic hallmark of their approach was an augmentation of mitochondrial routing by adding MTS and nuclear export signal (NES) domains instead of only MTS to basic mtDNA targeted amino acids sequence of ZFP [174]. To test the efficacy of site-specific alteration of mtDNA the authors combined a ZFP with the easily assayed DNA-modifying activity of hDNMT3a methylase. Expression of the mutation-specific chimeric methylase resulted in the selective methylation of cytosines adjacent to the mutation site. Interestingly, targeted mtDNA methylation itself can be an additional option to fight MD and mitochondrial dysfunction [175].

The next step was the design of mitochondria-targeted TALEN (mito-TALEN). In 2013 Bacman and colleagues showed irreversible elimination of mtDNA carrying major deletion m.8483_13459del4977 or pathogenic point mutation m.14459G > A in patient-derived cell cultures [176]. The similar strategy was next successfully tested in animal tissue cultures [177, 178], murine oocytes [179] and even plants [180–182].

For mitochondrial targeting, a few modifications of TALEN were proposed. Naoki Yahata and colleagues showed a therapeutic shift of heteroplasmy in human induced pluripotent stem cells (iPSCs) carrying m.13513G > A mutation of mtDNA after treatment by G13513A-mpTALEN conjugated with MTS (ATP5B7 or Cox8) [183]. Another modification, the replacement of nuclease domain FokI by the smaller one I-TevI resulted in increased mitochondrial permeability of TALE-I-TevI complex. This modified version was shown to shift heteroplasmy in patient-derived cybrids harboring different levels of the m.8344A > G mtDNA point mutation, associated with myoclonic epilepsy with ragged-red fibers (MERRF) [184].

Moreover, mitoTALEN was also used for the revelation of causative mechanisms leading to a “common deletion”, the most frequent aberrancy of the mitochondrial genome, involving a 4,977-bp region flanked by 13-bp repeats [185].

Finally, programmable nucleases were also tested in vivo in animal models. MitoTALEN, delivered by AAV9 vector was shown to effectively reduce the levels of mutant mtDNA and recovery of tRNA^Ala^ in a murine model of heteroplasmic MD-associated mutation [186]. Similarly, in mice with heteroplasmic pathogenic mutation m.5024C > T displaying typical characteristics of classic mitochondrial disease [187], AAV delivery of mitochondrially targeted zinc finger-nucleases (mtZFNs) induced elimination of mutant mtDNA and attenuation of biochemical phenotypes [188].

##### CRISPR/Cas

Unfortunately, there are still not enough studies testing CRISPR/Cas9-based approach for shift of heteroplasmy and results of existing are mostly controversial [189]. Whereas some authors reported CRISPR/Cas9 might be efficiently translocated into mitochondria [99, 168], others report its failure [190]. Possible reasons for the low effectiveness could be related to the low permeability of Cas9 nuclease. Because of its large size and low positive charge, Cas9, even containing MTS, poorly penetrates into mitochondria, and, having penetrated, has a destructive effect on them [191].

Moreover, some concerns about unfeasibility of RNA component of CRISPR/Cas9 to transfer into mitochondria questioned the applicability of this approach even more. To overcome these limitations, some authors adapted CRISPR/Cas components by appending the fragments of “mitochondria recognizable” motifs to RNA and proteins.

In general, these are the same strategies which were used to gain mitochondrial permeability of ZFN, TALEN and antireplicative machines. For instance, Loutre et al. designed CRISPR/Cas9 addressed to mitochondria (mito-CRISPR/Cas9) by adding *COX8A*-derived MTS to Cas9 and hairpin structures to sgRNA (FD-RNA). This system effectively shifted heteroplasmy in Kearns Sayre Syndrome cybrids. Interestingly, in this study, it turned out, that sgRNA molecules lacking the import determinants also penetrated to mitochondria, suggesting that own hairpins of sgRNA can substitute them [192]. Similar strategy was utilized by Hussain and colleagues by adding the RNA transport-derived stem loop element (RP-loop), naturally guiding RNA into mitochondria, to the sgRNA [193]. In combination with MTS-harboring Cas9, this complex was effectively targeted to mitochondria resulting in the decrease of mtDNA copy number in the cells with 11205G variant in their *ND4* sequence [193].

Antón et al. [191] conducted comparative trials of different modified Cas and revealed the most effective mitochondrial import for SaCas9 (*Staphylococcus aureus* Cas9), LbCas12a (*Lachnospiraceae bacterium* Cas12) and AsCas12a versions (*Acidaminococcus sp.* Cas12). Whereas Cas9 comprises two nuclease domains which generate blunt-ended DSBs of DNA [194, 195]. Cas12a, contains a single endonuclease domain that cleaves the two DNA strands in turn, resulting in a staggered DSB with a 5′ overhang [196]. Additionally, in this work MTS derived from Su9 (*Neurospora crassa* ATPase subunit 9) and mammalian ATG4D were shown to be more efficient to guide Cas isoforms to mitochondria [191].

In general, mito-CRISPR/Cas is a rapidly improving technology. Thus, CRISPR/Cas is a promising tool for shift of heteroplasmy, especially because of its adjustability and simplicity. However, to date therapeutic applicability of mito-CRISPR/Cas for mtDNA editing needs to be confirmed in further studies.

##### Other nucleases

Other nucleases which have been tested for shift of heteroplasmy are summarized in Table 3. All listed nucleases are non-programmable.

**Table 3:** Nucleases, proposed for elimination of mutant mtDNA

Nuclease	Brief description	Target	Reference
mitoTev‐TALE	Monomeric nuclease derived from T4 phage (I‐TevI). Smaller than TALEN and hence more suitable for package its coding gene into viral vectors	Programmable	[184]
PstI	Bacterial PstI endonuclease was recoded to optimize expression in mammalian cells and cloned downstream to MTS. Human mtDNA harbours two restriction sites for PstI (at positions 6914 and 9024)	Positions 6914 and 9024 of human mtDNA	[173]
Smal	Smal gene from *Serratia marcescens* appended by MTS sequence cloned from pCoxIV of *Saccharomyces cerevisiae*	T8399G mutation in NARP	[197]
Mito-ApaLI-HA	Synthetic ApaLI endonuclease from *Acetobacter pasterianus* added by MTS coding sequence. For immunological detection of ApaLI, a hemagglutinin epitope tag was added to its C terminus	Specific site in mtDNA of BALB mice	[198]

### mtDNA editing

As previously described, genome editing systems can be successfully delivered into mitochondria. Theoretically, as mitochondria are not able to repair DSBs in mtDNA, editing of the mitochondrial genome with the use of classic methods based on homological reparation seems scarcely feasible. Nevertheless, Bian and colleagues provided in vitro and in vivo evidences that mtDNA could be repaired through the mito-CRISPR/Cas9 system via homological recombination. In brief, an exogenous single-stranded DNA with short homologous arm was imported in the mitochondria of human cells. Moreover, the construction was knocked into the targeting loci of zebrafish, and this mutagenesis was steadily transmitted to F1 generation [168]. Obviously, the ability and versatility of homological reparation of mtDNA after DSBs needs to be confirmed in further studies, but to date this is one of the most promising strategies for the treatment of MD.

Meanwhile, another powerful approach to repair mtDNA mutations was proposed. Apparently, the novel strategy of programmable base edition without DSBs provides the precise tool to introduce petite changes in mtDNA [199–201]. Base edition is a form of genome editing that enables direct, irreversible conversion of one base pair to another at a target genomic locus without requiring double-stranded DNA cleavage, homology-directed repair processes, or donor DNA templates [202]. For this conversion two base editors could be used: cytosine base editor, which chemically converts a cytosine–guanine (C–G) base pair into a thymine–adenine (T–A) and adenine base editor, chemically transforming A–T to G–C base pairs. Base editors require one of the classical genome editing systems (CRISPR, TALE etc.) for guiding to target loci. Because deaminases can operate only on single-stranded nucleic acids [203] CRISPR, as machinery able to unwind double-stranded DNA, is usually utilized for base edition.

However, difficulties with mitochondrial addressing of sgRNA aimed Mok et al. to engineer non-CRISPR system which is able to operate with unwound dsDNA. The authors fused MTS-containing TALE and DddA, toxicity-lack derivative of interbacterial toxin catalyzing the deamination of cytidines within dsDNA. The resulting non-toxic RNA-free DddA-derived cytosine base editors was shown to catalyse C•G-to-T•A conversions in human mtDNA with high target specificity and product purity. This system was challenged for editing 5 mitochondrial genes: *MT-ND1*, *MT-ND2*, *MT-ND4*, *MT-ND5* and *MT-ATP8* in HEK293T cells. Depending on the sequence, the conversion efficiency of CG to TA reached 50% [204].

The same DddA-TALE machinery was further utilized for the development of the murine models of MD by Lee and colleagues. Two mutations of the *ND5* gene were successfully recapitulated into the mice: silent mutation, m.G12918A causing multiple human MD, and m.C12336T that incorporates a premature stop codon at the 199th position of the ND5 protein. Notably, it was also shown that mtDNA heteroplasmy induced by DdCBEs in one-cell stage zygotes can be maintained throughout the development and differentiation and transmitted to the next generation [205].

**Fig. 4:**
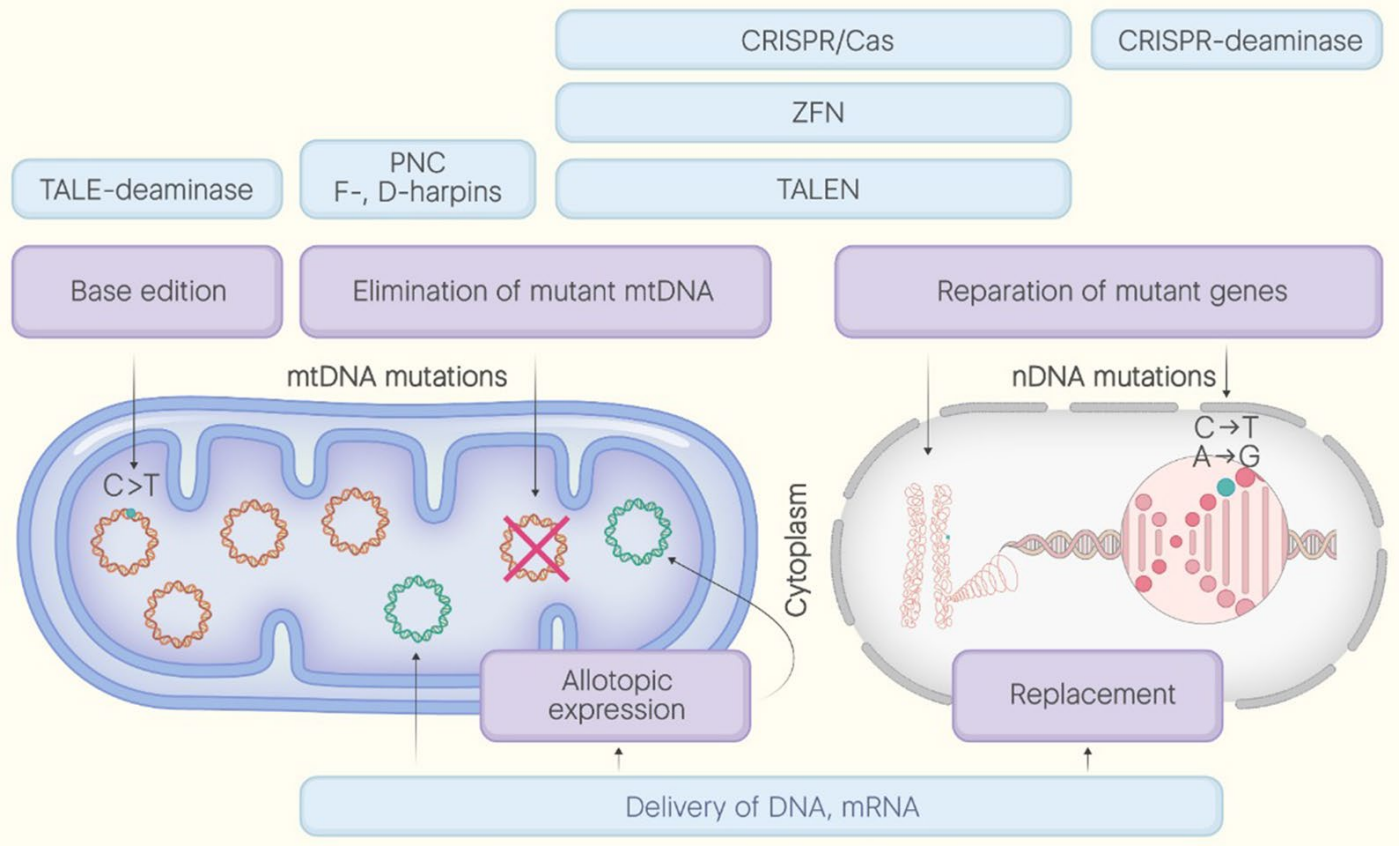
Multiple GT approaches for the treatment of MD. Existing approaches enable targeting both nuclear(n) or mitochondrial(mt) DNA. In general, possible interventions may be divided into the following strategies: (1) Reparation of mutant genes (including base edition); (2) Elimination of mutant mtDNA; (3) Replacement (including allotopic expression).**1**. Reparation of mutant nuclear genes can be performed with the use of programmed nucleases (TALEN, ZFN, CRISPR/Cas). A modified version of CRISPR (CRISPR-deaminase) can be utilized for base edition (introduction of single nucleotide substitutions) in nuclear genome whereas modified version of TALEN (TALEN-deaminase) can be utilized for base edition in the mitochondrial genome. **2**. In order to eliminate mutant mtDNA, double-stranded breaks induced by programmable nucleases TALEN, ZFN, CRISPR/Cas, can be used. Another option is application of antireplicative machines FD-RNA or peptide nucleic acids (PNA).**3**. The function of altered gene may also be compensated by its wild type copy delivered into mitochondria or nucleus. In the case of replacement of mitochondrial genes, sequence can be allotopically expressed in the nucleus, but only after reprogramming in mitochondrial genetic code and appending mitochondrial-targeting sequence

Thus, although quite laborious, the DddA-TALE method provides high efficiency of mitochondrial genome editing. The main GT approaches for the treatment of MD are presented in Fig. 4.

## Perspectives and difficulties

Unfortunately, the issues of the treatment of MD are still mostly up-in-the-air. Although only emerging, GT is the most promising avenue to treat MD, especially on the background of modest clinical efficacy of classical pharmacotherapy. In terms of applicability, modern GT approaches are quite close to successful treatment of those MD which are caused by nDNA mutations. However, nDNA mutations are found only in 15% of all patients struggling MD, whereas the prevalent majority of patients carry mutations in the mitochondrial genome [206].

Because of the major diversity of mutations associated with MD, all the emerging GT strategies could take an important place. To date some of them are close to the clinical application, but some–are still in the beginning stages of development. Largely, the strategies for a shift of heteroplasmy and edition of mtDNA are far from therapeutic implementation, although move by leaps and bounds. At the same time, because of its more solid background, the replacement GT approach apparently can advance to clinical application in the near future. Already, GT based on AAV-mediated delivery of the *ND4* with MTS for the treatment of LHON caused by a mutation in the mitochondrial genome has reached an advanced stage of clinical trials.

Additionally, there are also a few possible strategies we did not focus on in this paper. In this review, we did not include approaches based on antisense oligonucleotides (ASN). ASN is another promising strategy for manipulating mutant genes, but it is not GT in terms of FDA. Because of its ability to complementarily bind to a target site in pre-mRNA ASN may serve to reprogram or disrupt splicing of mutant genes resulting in a therapeutic effect [207]. Currently, ASN based therapies have been approved for the treatment of spinal muscular atrophy [208] and Duchenne muscular dystrophy [209]. This approach was also tested for silencing mtDNA [210–212] as well as splice correction or reducing the inclusion of a non-productive exon of *OPA1* [213, 214], disclosing its potential in treating MD.

Finally, interesting approaches based on the delivery of anti-apoptotic genes were proposed. For instance, recently Wassmer et al. utilized a murine model expressing mutant ND4 to test the potential of delivery of X-linked inhibitor of apoptosis (XIAP) to prevent retinal ganglion cell apoptosis and reduce disease progression. Authors reported significant amelioration of the disease course protecting the nerve fiber layer and optic nerve architecture [215]. XIAP is an inhibitor of caspases and apoptosis, and possible autophagy modulator [216]. As apoptosis and autophagy are key events caused by mitochondrial dysfunction, XIAP-based GT is a promising strategy for the treatment of MD, both nDNA and mtDNA-associated.

The remarkable thing in the issues of GT approaches for MD is that many data obtained are controversial. Although many breakthrough studies are reported there are even more studies reporting negative results when attempting reproduce them. In order to present state-of-the-art picture of mitochondrial GT we mostly focused on positive reports though mentioning some opposite results. Herein, we close our review with some critical concerns to not be misleading. In particular, even though some authors have demonstrated beneficial allotopic expression of mitochondrial genes, several lines of evidence demonstrate that the results showing import of allotopically expressed proteins are indeed artefactual [217]. The same is with technologies relying on mitochondrial import of RNA as the general view in the mitochondrial community is still that RNAs cannot enter mitochondria. In this regard, the feasibility of DSB-based editing of mitochondrial genome seems to be in especial need to be further challenged as it also does not correspond to existing view of mtDNA reparation. We believe that further studies may shed light on disputable questions of mitochondrial biology and open up brand new landscapes in the treatment of MD.

## Data Availability

Not applicable.

## Abbreviations

MD: Mitochondrial diseases
OXPHOS: Oxidative phosphorylation
mtDNA: Mitochondrial DNA
DCA: Dichloroacetate
GT: Gene therapy
siRNA: Small interfering RNA
shRNA: Short hairpin RNA
miRNA: MicroRNA
CRISPR/Cas: Clustered regularly interspaced short palindromic repeats / CRISPR-associated protein
ETC: Electron transport chain
MELAS: Mitochondrial Encephalopathy, Lactic Acidosis, and Stroke-like episodes
MIDD: Maternally inherited diabetes and deafness
ZFN: Zinc fingers nucleases
TALEN: Transcription activator-like effector nucleases
DQAsomes: Dequalinium-based liposome-like vesicles
rAAV: Recombinant adeno-associated viruses
IVT: Intravitreal
SC: Suprachoroidal
SR: Subretinal
MRIgFUS: Magnetic resonance-guide focused ultrasound
MTSs: Mitochondrial targeting sequences
*SOD2*: Superoxide dismutase 2 gene
*TYMP*: Thymidine phosphorylase gene
*Slc25a46*: Mitochondrial fusion-fission-related gene solute carrier family 25 member 46
*OPA1*: Dynamin-like 120 kDa protein
*NDUFS4*: NADH dehydrogenase (ubiquinone) iron-sulfur protein 4 gene
*Fdxr*: Ferredoxin reductase gene
*ETHE1*: Ethylmalonic encephalopathy gene
SDO: Sulfur dioxygenase
LHON: Leber hereditary optic neuropathy
RGC: Retinal ganglion cells
NARP: Neuropathy, ataxia and retinitis pigmentosa
