# Correction: Current advances in gene therapy of mitochondrial diseases

**DOI:** 10.1186/s12967-023-03915-z

**Published:** 2023-02-08

**Authors:** Vladislav O. Soldatov, Marina V. Kubekina, Marina Yu. Skorkina, Andrei E. Belykh, Tatiana V. Egorova, Mikhail V. Korokin, Mikhail V. Pokrovskiy, Alexey V. Deykin, Plamena R. Angelova

**Affiliations:** 1 Core Facility Centre, Institute of Gene Biology, Russian Academy of Sciences, Moscow, Russia; 2 Department of Pharmacology and Clinical Pharmacology, Belgorod State National Research University, Belgorod, Russia; 3 Department of Biochemistry, Belgorod State National Research University, Belgorod, Russia; 4 Dioscuri Centre for Metabolic Diseases, Nencki Institute of Experimental Biology, Polish Academy of Sciences, Warsaw, Poland; 5 Laboratory of Modeling and Gene Therapy of Hereditary Diseases, Institute of Gene Biology, Russian Academy of Sciences, Moscow, Russia; 6 Laboratory of Genome Editing for Biomedicine and Animal Health, Belgorod State National Research University, Belgorod, Russia; 7 Department of Clinical and Movement Neurosciences, UCL Queen Square Institute of Neurology, London, UK

**Correction to: Journal of Translational Medicine (2022) 20:562** **https://doi.org/10.1186/s12967-022-03685-0**

Following publication of the original article [1], we have been notified that corresponding author affiliations should be as per:

- Vladislav O. Soldatov^1,2,6*^
